# A Sri Lankan child with 49,XXXXY syndrome

**DOI:** 10.4103/0971-6866.73413

**Published:** 2010

**Authors:** Vajira H. W. Dissanayake, Palinda Bandarage, Christeen R. J. Pedurupillay, Rohan W. Jayasekara

**Affiliations:** 1 Human Genetics Unit, Faculty of Medicine, University of Colombo, Colombo, Sri Lanka; 2 Asiri Centre for Genomic and Regenerative Medicine, Asiri Surgical Hospital, Colombo, Sri Lanka

**Keywords:** Ambiguous genitalia, sex chromosome aneuploidy, XXXXY syndrome

## Abstract

Pentasomy 49,XXXXY is a rare sex chromosome disorder usually presenting with ambigous genitalia, facial dysmorphism, mental retardation and a combination of cardiac, skeletal and other malformations. The incidence of the condition is estimated to be 1 in 85,000 male births. Previously, this condition was identified as a Klinefelter variant. The condition is suspected in a patient, by a combination of characteristic clinical findings, and the diagnosis is confirmed by chromosome culture and karyotyping. In the case we report here, the main presentation of ambiguous genitalia led to a suspicion of a sex chromosome aneuploidy which was subsequently confirmed by chromosomal analysis.

## Introduction

Pentasomy 49,XXXXY is a rare sex chromosome polysomy, clinically expressed as a combination of mental retardation, facial dysmorphism, genital, cardiac, and skeletal malformations. The incidence of the condition is estimated to be 1 in 85,000 male births.[1] It was originally described by Fraccaro and colleagues in 1960.[2] The case we report here, to the best of our knowledge, is the first such case reported from SriLanka.

## Case Report

A baby boy who was noticed to have ambiguous genitalia at birth was referred for genetic studies. He was the first baby born to a non-consanguineous couple (a 21-year-old woman and a 29-year-old man). The pregnancy had been uneventful till delivery. The baby has been delivered at 38 weeks of gestation by lower segment cesarean section due to the head not being engaged. The birth weight was 2.150 kg and he had been kept in the special care baby unit due to grunting and a pan systolic cardiac murmur noticed at birth. The child had developed head control by the age of 3 months and showed social smile at 10 weeks of age. The other examination findings were: brachycephaly, mild upward slant of the eyes, flat nasal bridge, bilateral clinodactyly and micropenis (Both testis were in well formed scrotal sacs. The urethral opening was at the normal position on the tip of the penis). 2D echocardiogram showed situs solitus and levocardia with a fenestrated interatrial septum leading to a left to right shunt, mild dilatation of right atrium and right ventricle, mild muscular VSD with left to right shunting, trivial aortic regurgitation, and valvular pulmonary stenosis. Ultrasound scan of the abdomen showed bilateral hydroceles.

**Figure 1:**
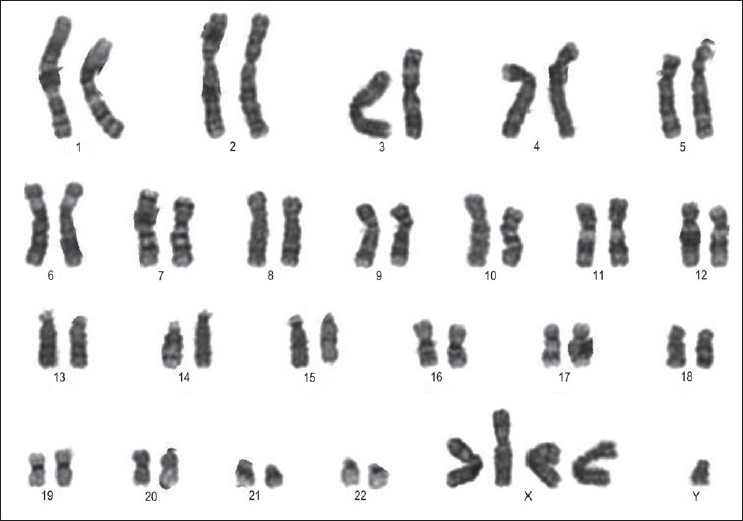
Karyogram showing the 49,XXXXY karyotype

Chromsome culture and karyotyping showed a karyotype of 49,XXXXY [Figure 1].

## Discussion

Although initially 49,XXXXY pentasomy was considered a variant of Klinefelter syndrome, it is currently recognized as a separate clinical entity distinguished by facial features, multiple skeletal and cardiac defects and short stature. A 49,XXXXY karyotype is thought to arise from maternal non-disjunction which occurs during both meiosis I and meiosis II. This produces a secondary oocyte with four X chromosomes, which, when fertilized by a Y chromosome bearing sperm, results in an embryo with 49,XXXXY syndrome.[3]

Characteristic clinical features of 49,XXXXY syndrome are the triad of mental retardation, radioulnar synostosis, and hypogonadism. There are a range of other associated phenotypic features such as low birth weight, slow growth with retarded bone age, craniofacial anomalies, abnormal genitals, widely spaced nipples, cardiac deformities, and skeletal abnormalities. Facial dysmorphism is characterized by a full round face, hypertelorism, telecanthus, and upslanted palpebral fissures.

In this case, the genital abnormalities were the main features which led to a suspicion of a sex chromosome aneuploidy that was confirmed by chromosomal analysis. There are previous reports of the condition diagnosed in babies presenting with genital abnormalities at birth.[4]

The prognosis of these children depends on the extent of severity of the condition while the management mandates a multidisciplinary approach with pediatric endocrinology, pediatric surgery, orthopedics, psychiatry, and clinical genetic evaluations.

## References

[1] Visootsak J, Graham JM (2006). Klinefelter syndrome and other sex chromosomal aneuploidies. Orphanet J Rare Dis.

[2] Fraccaro M, Kaijser K, Lindsten J (1960). A child with 49 Chromosomes. Lancet.

[3] Villamar M, Benitez J, Fernández E, Ayuso C, Ramos C (1989). Parental origin of chromosomal nondisjunction in a 49, XXXXY male using recombinant-DNA techniques. Clin Genet.

[4] Ng SF, Boo NY, Wu LL, Shuib S (2007). A rare case of ambiguous genitalia. Singapore Med J.

